# Discovery of Room-Temperature Topological Insulators in Functionalized Group VA-VA Binary Monolayers: A First-Principles Investigation

**DOI:** 10.3390/ma18215017

**Published:** 2025-11-04

**Authors:** Clement Ding, Xuan Luo

**Affiliations:** National Graphene Research and Development Center, Springfield, VA 22151, USA; xluo@ngrd.org

**Keywords:** topological insulators, first-principles calculation, binary monolayers, functionalization, band structure, room-temperature

## Abstract

Topological insulators and semimetals are necessary to realize quantum computing and spintronics. We use first-principles calculations to investigate the atomic structure, electronic band structure, and Z_2_ invariants of four sets of pure and functionalized buckled hexagonal monolayers that are promising candidates for topological nature: BiAs, AsP, SbAs, BiSb, and functionalized monolayers BiAsX_2_, AsPX_2_, SbAsX_2_, and BiSbX_2_ (X = H, O, S). Our results show that BiAsO_2_, BiAsS_2_, AsPO_2_, SbAsO_2_, SbAsS_2_, BiSbH_2_, BiSbO_2_, and BiSbS_2_ are topological insulators with small SOC-induced band gaps ranging from 0.05 to 0.37 eV. Further, we propose AsPS_2_ to be a topological semiconductor. Topological insulators stand on the boundary of induction and conductance and are crucial in realizing quantum computers. The room-temperature topological insulators predicted here will have promising impacts in quantum computing, nanoelectronics, and spintronics.

## 1. Introduction

Topological materials, specifically topological insulators (TIs) and topological semimetals (TSMs), have been shown to have novel surface states that are different from their bulk states [1]. TIs are known to be insulating in the bulk with gapless edge states [2] due to the inversion of the conduction and valence bands by strong spin orbit coupling (SOC) [3]. These surface states have spin-momentum locking, which allows for exotic electron transport properties [4] and makes TIs valuable in spintronics. TIs are also valuable in thermoelectrics for their unique combination of an insulating bulk with conducting surface states. In the past decade, there has been an explosion of research into new TSMs and identifying and categorizing TSMs into Dirac, Weyl, and many other categories [5,6]. TSMs show great potential for quantum computing [7] and spintronics [8]. TIs and TSMs both have robust boundary states resistant to perturbations [9], which is crucial for protecting delicate quantum systems from unwanted noise.

Many materials have been theorized to be TIs, but few have been confirmed experimentally. The first TI was the CdTe/HgTe/CdTe quantum well [10,11], which consisted of a thin layer of HgTe between two layers of CdTe. Soon after, the first 3D TI was experimentally confirmed: Bi1_−x_Sb_x_ [10,12,13]. Well-known 3D TIs include Bi_2_Se_3_, Bi_2_Te_3_, and Sb_2_Te_3_ [2,12]. However, 3D TIs are relatively difficult to use in spintronics and other applications due to degradation when scaled down [14], and thus researchers have shifted their focus to 2D topological materials candidates. Theoretically predicted and experimentally verified 2D TIs include bismuthene [15,16] and Na_3_Bi [15,17], which have the largest topological gaps to date [15]. Recently, research has been focused on searching for room-temperature 2D TIs, which are crucial for room-temperature spintronics [18]. Additionally, the edge states of 2D TIs are one of the few places that Majorana fermions, crucial to quantum computing, can be observed [19].

In past decades, 2D monolayer topological material candidates have been the center of investigations both theoretically and experimentally. Single-element monolayer TIs include arsenene [6], antimonene [6], silicene [20], germanene [15,20,21], and stanene [20], which have all been experimentally confirmed. Multi-element monolayer TIs include the aforementioned bismuthene [15,16]. In particular, 2D binary monolayers are gaining attention, such as the experimentally confirmed group IIIA-VA binary monolayers GaBi, InBi, TlBi, TlAs, TlSb, and TlN [22]. However, these materials have small band gaps that are not significant at room temperature, necessitating further research for room-temperature TIs.

Most recently, the Group-VA family has attracted interest for their variety of allotropes with properties that make them suitable for electronics, spintronics, and energy devices [6]. 2D VA-VA binary monolayers have attracted great attention for their stable structure and material properties [23,24,25,26,27,28,29,30,31,32,33]. The buckled honeycomb structures of BiAs, AsP, SbAs, and BiSb have all already been investigated for topological nature, with BiAs, SbAs, and BiSb all being strain-induced TIs [34,35,36]. We will briefly describe our reproduction of the results published in previous literature in Section 3.1. We did not calculate strain effects. Additionally, functionalization of binary monolayers is a well-known method for creating a topological phase transition [37].

To date, there is still a gap in the research of room-temperature topological insulators. We dive in to provide insight into room-temperature topological insulator candidates by pursuing functionalized Group VA-VA monolayers in order to realize a new direction for topological materials research. Our focus is on investigating: BiAs and functionalized BiAsX_2_ (X = H, O, S); AsP and functionalized AsPX_2_ (X = H, O, S); SbAs and functionalized SbAsX_2_ (X = H, O, S); and BiSb and functionalized BiSbX_2_ (X = H, O, S) for their atomic structure, electronic properties, and topological nature. We have reproduced pure BiAs, AsP, SbAs, and BiSb to prove the validity of our method. The novelty of our research is in the functionalized BiAsX_2_, AsPX_2_, SbAsX_2_, and BiSbX_2_ (X = H, O, S).

In Section 2, we detailed our methods to perform first-principles calculations. In Section 3, we present our results. We discuss and compare our results with other theoretical research in Section 3.1. Finally, our conclusion and directions for future research are found in Section 4.

## 2. Materials and Methods

We performed first-principles calculations based on Density Functional Theory (DFT) within the Generalized Gradient Approximation (GGA) in Perdew-Burke-Ernzerhof (PBE) format implemented in the ABINIT [38] 9.8.4 code. We use the Projector Augmented Wave (PAW) method to generate [39] pseudopotentials with the ATOMPAW 4.0.0.12 code [40]. Scalar-relativistic PAW pseudopotentials were used in all calculations. The electron configurations and radius cutoffs of the atoms used in current calculations are listed in Table 1.

Total energy calculations using self-consistent field (SCF) iterations were considered converged when the difference in total energy between two adjacent iterations was less than 1.0 × $10^{-10}$ Ha twice consecutively. The kinetic energy cutoff, Monkhorst-Pack *k*-point grid, and vacuum height were considered converged when the total energy difference between two neighboring datasets was lower than 1.0 × $10^{-4}$ Ha twice. These convergence criteria have been used in previous research [10,41] and proven to be reliable. The converged values for each structure are listed in Table 2.

The Broyden–Fletcher–Goldfarb–Shanno (BFGS) minimization algorithm was used for structural optimization. Each structure was considered fully relaxed once the maximum forces were less than 1.0 × $10^{-5}$ Ha/Bohr.

The electronic structures of each material were analyzed through band structure and fatband structure calculations. All band structure calculations were carried out using the following high-symmetry k-point circuit in the irreducible first Brillouin zone: Γ (0.0, 0.0, 0.0) → K (1/3, 2/3, 0.0) → M (1/2, 1/2, 0.0) → Γ (0.0, 0.0, 0.0).

We used the Wannier90 [42] 3.1.0 software package to track the evolution of the hybrid Wannier charge centers (HWCC), which has been used frequently and proven reliable in previous literature [10,41]. We used the Z2Pack [43,44] 2.2.1 software package to determine the Z_2_ invariant.

**Figure 1:**
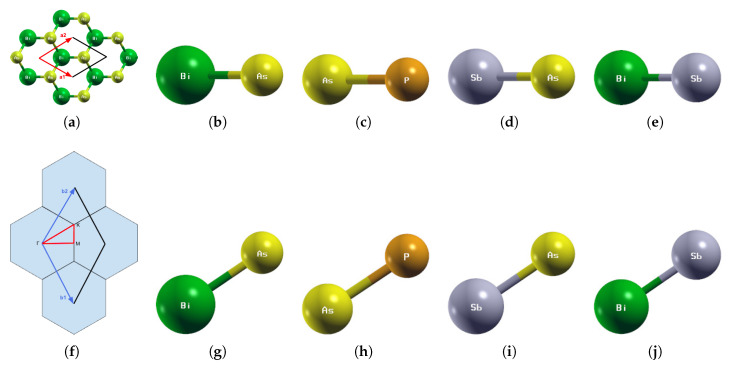
(**a**) The primitive vectors and (**f**) the brillouin zone of BiAs. Displayed here are the top (**b**–**e**) and side (**g**–**j**) views of a 1 × 1 × 1 supercell of the optimized atomic structures of (**b**,**g**) BiAs, (**c**,**h**) AsP, (**d**,**i**) SbAs, and (**e**,**j**) BiSb. Each atom is labeled with its atomic symbol. The green, yellow, orange, and gray atoms represent Bi, As, P, and Sb, respectively.

Our calculations are first-principle calculations, so no parameters were involved. The input data consists only of the atomic pseudopotentials and the lattice structure. We used the pure materials BiAs, AsP, SbAs, and BiSb to prove the validity of our methodology, which serves as justification for the reliability of our other results and provides reproducibility (Figure 1).

## 3. Results and Discussion

We investigated Group-VA monolayers BiAs, AsP, SbAs, and BiSb, as well as functionalized monolayers BiAsX_2_, AsPX_2_, SbAsX_2_, BiSbX_2_ (X = H, O, S).

We calculated the optimized atomic structure, the band structures with and without SOC, the fatbands, and the Z_2_ invariant.

### 3.1. Pure BiAs, AsP, SbAs, BiSb

**Figure 2:**
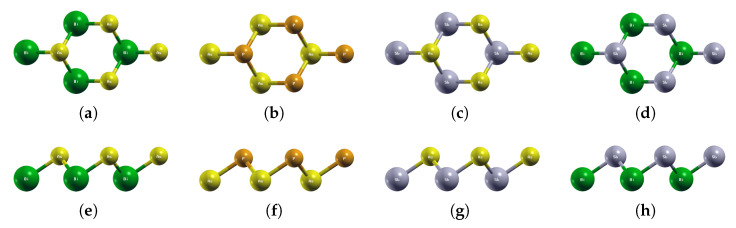
Optimized atomic structures of BiAs, AsP, SbAs, and BiSb. Displayed here are the (**a**–**d**) top and (**e**–**h**) side views of a 2 × 2 × 1 supercell of (**a**,**e**) BiAs; (**b**,**f**) AsP; (**c**,**g**) SbAs; (**d**,**h**) BiSb. Each atom is labeled with its atomic symbol. The green, yellow, orange, and gray atoms represent Bi, As, P, and Sb, respectively.

The optimized atomic structures of pure BiAs, AsP, SbAs, and BiSb are shown in Figure 2. All four monolayers are buckled honeycomb structures, similar to Boron Nitride from a top view but appearing as a zigzag pattern from the side view. Each structure has two atoms per unit cell. The lattice parameters of the above four pure monolayers are listed in Table 3. The current lattice constants and buckling heights are consistent with previous first-principles calculations [24,28,31,35,45,46,47,48].

**Figure 3:**
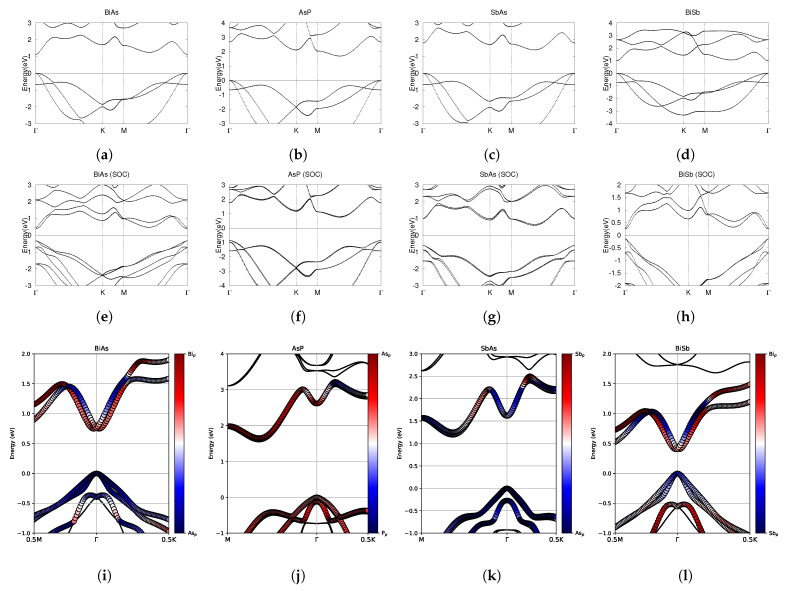
Electronic band structures of pure monolayers BiAs, AsP, SbAs, and BiSb. (**a**–**d**) The top row displays the bands without SOC. (**e**–**h**) The middle row displays the bands with SOC. (**i**–**l**) The bottom row shows the fatbands, with SOC. The fermi energy level position from the DFT calculation is set to 0.

The electronic band structures are clearly displayed in Figure 3. As shown in the first and second rows of Figure 3, all four materials have their valence band maximum (VBM) at the Γ point, both with and without SOC effects. All four electronic band structures show double degeneracies in both the valence and conduction band at the Γ high-symmetry point with SOC. BiAs has a direct band gap at Γ, and an indirect band gap with SOC due to band splitting. In BiAs, we have calculated a band gap of 0.75 eV, comparable to the previously calculated value of 0.70 eV [45]. AsP has conduction band minimum CBM between the M and Γ points both with and without SOC, as shown in Figure 3a,e. AsP has an indirect band gap of 1.70 eV, which is smaller than the previously calculated value of 1.82 eV [46]. The difference may be due to the semi-empirical DFT-D2 correction scheme used in previous calculations [46]. Similarly to AsP, SbAs has CBM between the M and Γ points both with and without SOC, as shown in Figure 3c,g. SbAs has an indirect band gap of 1.39 eV, and 1.20 eV with SOC, which is comparable to previous calculated values of 1.47 eV and 1.27 eV with SOC [35]. In BiSb, we have calculated a band gap with and without SOC of 0.40 and 1.00 eV, comparable to previously calculated values of 0.37 [47] and 0.36 eV [48] with SOC and 0.95 [47] and 0.96 eV [48] without SOC.

It is well known that larger atoms, especially Bismuth, will show stronger SOC effects [49]. The current calculation found that molecular structures with higher atomic mass experienced greater SOC effects (SOC reduces the band gap of BiSb by 0.60 eV, compared to a 0.09 eV band gap reduction for AsP), as shown in Table 3.

Investigating the fatbands in the third row of Figure 3, BiAs seems to have some inversion, especially in the conduction bands. The valence bands are dominated by As-4p orbitals, with some influence from Bi-6p orbitals. AsP has valence bands and CBM entirely dominated by the As-4p orbitals, as shown in Figure 3j and does not show significant band inversion. SbAs also has valence bands and CBM entirely dominated by As-4p orbitals, as shown in Figure 3k, and does not show significant band inversion. BiSb seems to have some inversion, similar to BiAs. The valence bands change from 6p orbital dominated to 5p orbital dominated around the Γ point.

All four band structures are in agreement with previous literature [25,35,45,47,48], which confirms the validity of our band structures.

**Figure 4:**
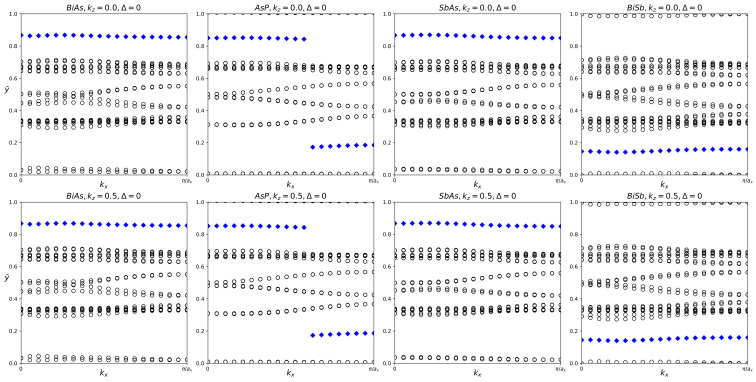
Hybrid Wannier charge centers for pure BiAs, AsP, SbAs, and BiSb monolayers. The open circles represent hybrid Wannier charge centers. The first row is at the surface k_z_ = 0. The second row is at the surface k_z_ = 0.5. The Δ indicates the Z_2_ invariant—0 represents trivial topology, while 1 represents nontrivial topology.

Although none of the four fatbands show clear band inversion, it is still worthwhile to investigate their Z_2_ indices. Tracking the movement of the Hybrid Wannier Change Centers reveals that all four pure structures have Z_2_ = 0, as displayed in Table 3 and Figure 4.

As previously stated in Section 1, BiAs, SbAs, and BiSb are already proven to be strain-induced topological insulators [34,35,36]. Our calculations of BiAs, AsP, SbAs, and BiSb are carried out at 0 pressure to replicate former work and show that our methodology produces results in agreement with previous literature.

### 3.2. BiAsX_2_ (X = H, O, S)

**Figure 5:**
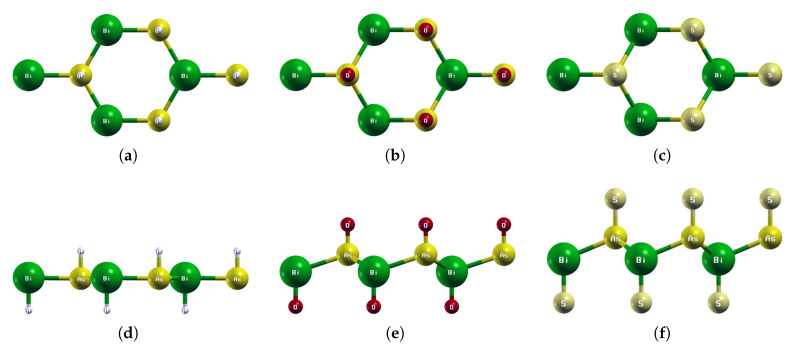
Optimized atomic structures of BiAsX_2_ (X = H, O, S). Displayed here are the (**a**–**c**) top and (**d**–**f**) side views of a 2 × 2 × 1 supercell of (**a**,**d**) BiAsH_2_; (**b**,**e**) BiAsO_2_; (**c**,**f**) BiAsS_2_. Each atom is labeled with its atomic symbol. The green, yellow, white, red, and grayish-yellow atoms represent Bi, As, H, O, and S, respectively.

**Figure 6:**
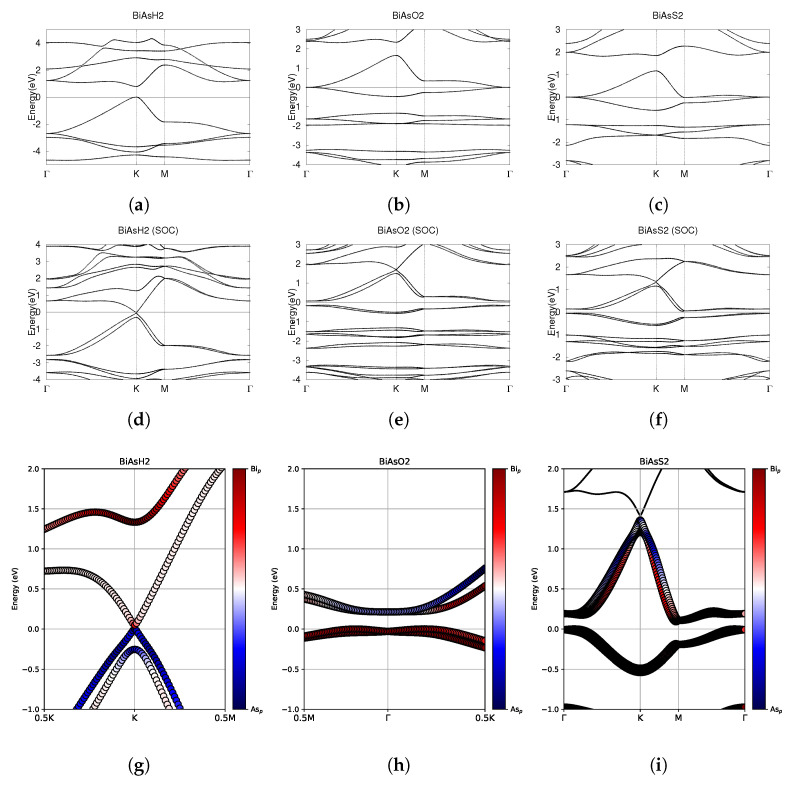
Electronic band structures of functionalized monolayers BiAsH_2_, BiAsO_2_, and BiAsS_2_. (**a**–**c**) The top row displays the bands without SOC. (**d**–**f**) The middle row displays the bands with SOC. (**g**–**i**) The bottom row shows the fatbands, with SOC. The fermi energy level position from the DFT calculation is set to 0.

The optimized atomic structures of BiAsX_2_ (X = H, O, S) are shown in Figure 5. All four monolayers are buckled honeycomb structures, appearing as a zigzag pattern from the side view. Each structure has four atoms per unit cell. The lattice constants, buckling heights, and electronic band structures of the functionalized BiAsX_2_ monolayers are shown in Table 4 and Figure 6.

Notably, the side view of BiAs_2_ as shown in Figure 5d reveals that BiAsH_2_ buckles inwards rather than outwards like BiAsO_2_ and BiAsS_2_. BiAsH_2_ also has an extremely small buckling height of 0.04Å compared to 0.96 Å for BiAsO_2_ and 1.15 Å for BiAsS_2_.

The band structure without and with SOC, as well as the fatbands, are shown in Figure 6. BiAsH_2_ is a semiconductor with a direct band gap of 0.79 eV at the K point. BiAsH_2_ uniquely experiences very strong SOC in the conduction band. The VBM and CBM do not become degenerate at the K point with or without SOC. There is a very small but nonzero direct band gap of 0.05 eV with SOC, as noted in Table 4. Figure 6b shows that BiAsO_2_ has CBM and VBM degenerate at the Γ point, forming a Dirac point. As shown in Figure 6c, the CBM of BiAsS_2_ is at the M point and is below the VBM, suggesting that BiAsS_2_ is semimetallic. In both BiAsO_2_ and BiAsS_2_, SOC effects break the degeneracy at the Γ point, which is a well known indicator of significant topological nature. After SOC, BiAsO_2_ has an indirect band gap of 0.21 eV, and BiAsS_2_ has an indirect band gap of 0.09 eV.

Investigating the fatbands in the third row of Figure 6, BiAsH_2_ shows little evidence of band inversion. The conduction bands of BiAsH_2_ are entirely dominated by Bi-6p orbitals and the valence bands are solely dominated by As-4p orbitals. The bands around the fermi level in BiAsO_2_ and BiAsS_2_ seem to be largely dominated by the Bi-6p orbitals, with one conduction band in both BiAsO_2_ and BiAsS_2_ being dominated by As-4p orbitals.

**Figure 7:**
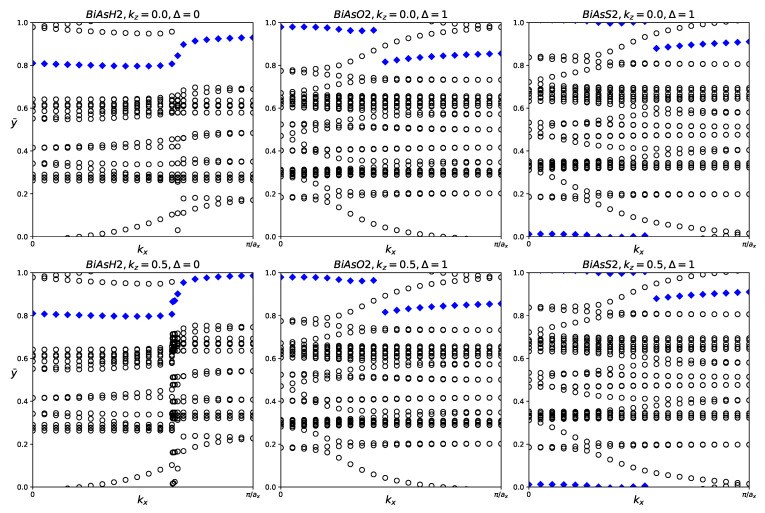
Hybrid Wannier charge centers for functionalized BiAsX_2_ monolayers (X = H, O, S). The open circles represent hybrid Wannier charge centers. The first row is at the surface k_z_ = 0. The second row is at the surface k_z_ = 0.5. The Δ indicates the Z_2_ invariant—0 represents trivial topology, while 1 represents nontrivial topology.

Although the fatbands for BiAsO_2_ and BiAsS_2_ appear to lack band inversion, it is still worthwhile to examine the Z_2_ indices of all three materials. The results of tracking the evolution of the hybrid Wannier charge centers are shown in Figure 7. BiAsH_2_ has Z_2_ = 0, indicating trivial topology. BiAsO_2_ and BiAsS_2_ both have Z_2_ = 1, which confirms that both materials are topologically nontrivial.

BiAsH_2_ is a trivial semiconductor, while BiAsO_2_ and BiAsS_2_ are small band gap topological insulators.

### 3.3. AsPX_2_ (X = H, O, S)

**Figure 8:**
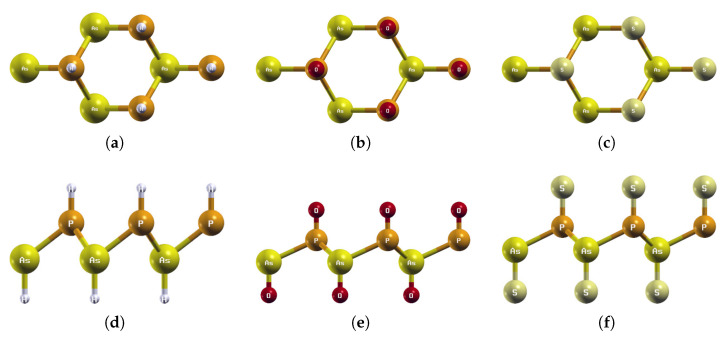
Optimized atomic structures of AsPX_2_ (X = H, O, S). Displayed here are the (**a**–**c**) top and (**d**–**f**) side views of a 2 × 2 × 1 supercell of (**a**,**d**) AsPH_2_; (**b**,**e**) AsPO_2_; (**c**,**f**) AsPS_2_. Each atom is labeled with its atomic symbol. The yellow, orange, white, red, and grayish-yellow atoms represent As, P, H, O, and S, respectively.

**Figure 9:**
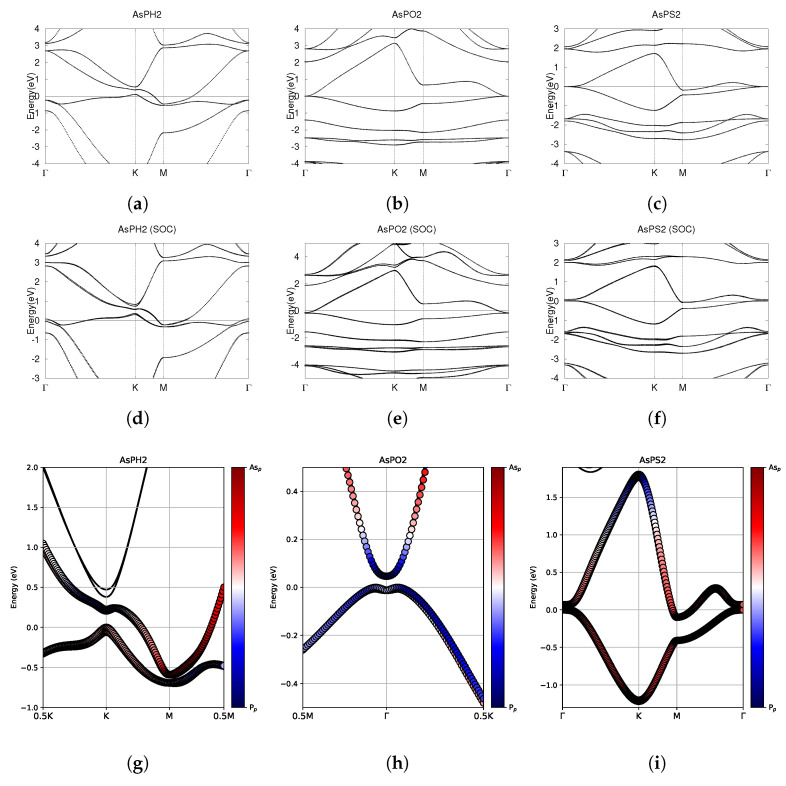
Electronic band structures of functionalized monolayers AsPH_2_, AsPO_2_, and AsPS_2_. (**a**–**c**) The top row displays the bands without SOC. (**d**–**f**) The middle row displays the bands with SOC. (**g**–**i**) The bottom row shows the fatbands, with SOC. The fermi energy level position from the DFT calculation is set to 0.

The optimized atomic structures of AsPX_2_ (X = H, O, S) are shown in Figure 8. All four monolayers are buckled honeycomb structures, appearing as a zigzag pattern from the side view. Each structure has four atoms per unit cell. The lattice constants, buckling heights, and electronic band structures of the functionalized AsPX_2_ monolayers are shown in Table 5 and Figure 9.

As shown in Figure 8, AsPH_2_ buckles outwards like AsPO_2_ and AsPS_2_. AsPH_2_ has a relatively large buckling height of 1.54 Å, compared to 1.09 Å for AsPO_2_ and 1.15 Å for AsPS_2_. Among all four H2-functionalized monolayers, AsPH_2_ is the only structure to buckle outwards. BiAsH_2_ has already been discussed in Section 3.2. SbAsH_2_ and BiSbH_2_ will be discussed later in Section 3.4 and Section 3.5, and are shown to buckle inwards in Figures 11d and 14d.

Investigating the electronic band structure, Figure 9a shows that AsPH_2_ has a negative indirect band gap, suggesting a semimetallic nature. Both with and without SOC, the CBM is at the M point and the VBM is at the K point. As shown in Figure 9b, AsPO_2_ has a Dirac point at the Γ point. When including SOC effects, the degeneracy at the Γ point is broken, indicating significant topology. After SOC, AsPO_2_ has a small indirect band gap of 0.05 eV. Figure 9c reveals that AsPS_2_ has a band crossing at the Γ point. The CBM is at the M point and crosses the Fermi level. The indirect negative band gap suggests that AsPS_2_ is a semimetal. Similarly to AsPO_2_, SOC breaks the degeneracy at the Γ point, indicating significant topological nature.

Investigating the fatbands in the third row of Figure 9, AsPH_2_ shows little evidence of band inversion. The fatbands for AsPO_2_ suggest possible band inversion, with the conduction band changing from being largely dominated by the As-4p orbital to the P-3p orbital. AsPS_2_ is largely dominated by As-4p orbitals. The conduction band is dominated by the P-3p orbitals at high energy levels.

**Figure 10:**
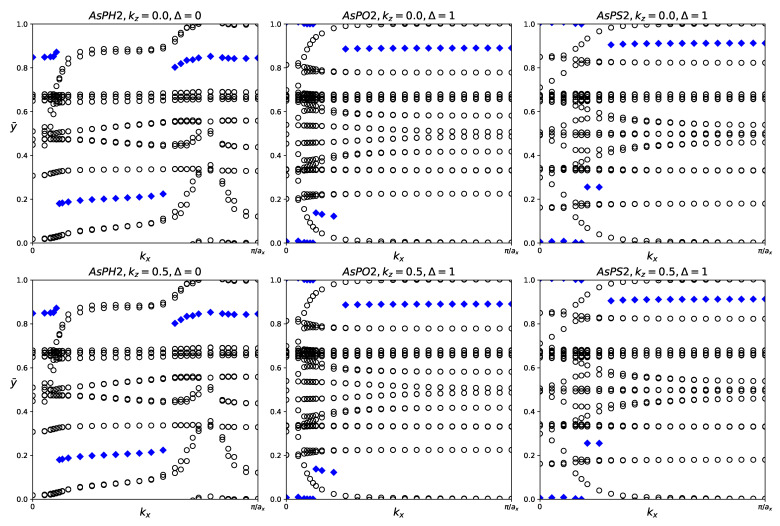
Hybrid Wannier charge centers for functionalized AsPX_2_ monolayers (X = H, O, S). The open circles represent hybrid Wannier charge centers. The first row is at the surface k_z_ = 0. The second row is at the surface k_z_ = 0.5. The Δ indicates the Z_2_ invariant—0 represents trivial topology, while 1 represents nontrivial topology.

Despite no conclusive evidence of band inversion in the fatbands, it is still worthwhile to investigate the z2 indices of all three structures. The evolution of the hybrid Wannier charge centers and the Z_2_ invariant are shown in Figure 10. The Z_2_ indices are also listed in Table 5. AsPH_2_ has Z_2_ = 0, indicating trivial topology. AsPO_2_ and AsPS_2_ both have Z_2_ = 1, indicating nontrivial topology.

AsPH_2_ is a trivial semiconductor, AsPO_2_ is a small band gap topological insulator, and AsPS_2_ is a topological semimetal.

### 3.4. SbAsX_2_ (X = H, O, S)

**Figure 11:**
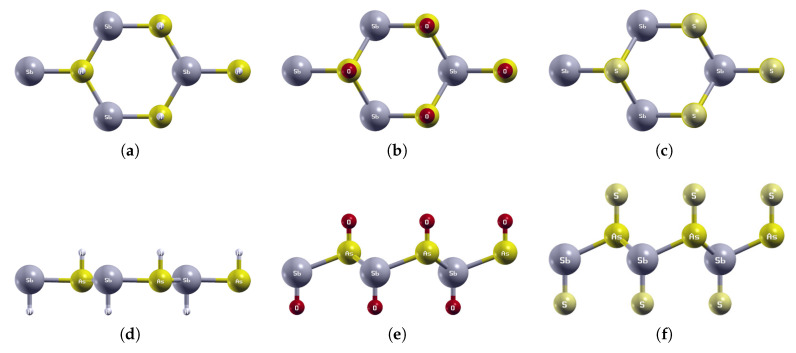
Optimized atomic structures of SbAsX_2_ (X = H, O, S). Displayed here are the (**a**–**c**) top and (**d**–**f**) side views of a 2 × 2 × 1 supercell of (**a**,**d**) SbAsH_2_; (**b**,**e**) SbAsO_2_; (**c**,**f**) SbAsS_2_. Each atom is labeled with its atomic symbol. The gray, yellow, white, red, and grayish-yellow atoms represent Sb, As, H, O, and S, respectively.

**Figure 12:**
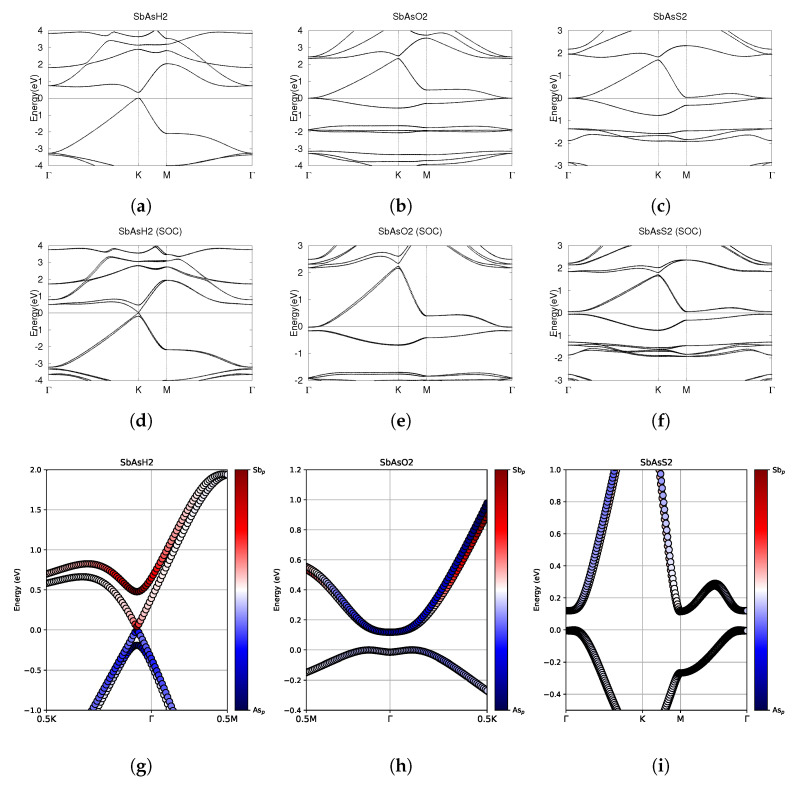
Electronic band structures of functionalized monolayers SbAsH_2_, SbAsO_2_, and SbAsS_2_. (**a**–**c**) The top row displays the bands without SOC. (**d**–**f**) The middle row displays the bands with SOC. (**g**–**i**) The bottom row shows the fatbands, with SOC. The fermi energy level position from the DFT calculation is set to 0.

The optimized atomic structures of SbAsX_2_ (X = H, O, S) are shown in Figure 11. All four monolayers are buckled honeycomb structures, appearing as a zigzag pattern from the side view. Each structure has four atoms per unit cell. The lattice constants, buckling heights, and electronic band structures of the functionalized SbAsX_2_ monolayers are shown in Table 6 and Figure 12.

As shown in Figure 11a, SbAsH_2_ buckles inwards. SbAsO_2_ and SbAsS_2_ buckle outwards. Similarly to BiAsH_2_, SbAsH_2_ has a very small buckling height of 0.06 Å compared to 1.05 Å for SbAsO_2_ and 1.20 Å for SbAsS_2_, as listed in Table 6.

Investigating the electronic band structure, Figure 12a,d reveals that SbAsH_2_ has a direct band gap at the K point both with and without SOC. Without SOC, SbAsH_2_ has a band gap of 0.35 eV. Similarly to BiAsH_2_ as shown in Figure 6a,d, SOC-induced spin splitting in the conduction band reduces the band gap of SbAsH_2_ to a very small value of 0.04 eV. SbAsO_2_ has CBM and VBM degenerate at the Γ point, forming a Dirac point. Including SOC effects breaks the degeneracy at Γ, indicating significant topological nature. With SOC, the conduction bands are twofold degenerate at the Γ point, as shown in Figure 12b. The valence bands are also twofold degenerate at the Γ point. After SOC, SbAsO_2_ has an indirect band gap of 0.12 eV, as listed in Table 6 and shown in Figure 12e. As shown in Figure 12c, SbAsS_2_ has CBM crossing the Fermi level at the M point. The conduction and valence bands are degenerate at the Γ point. This degeneracy is broken by SOC effects, suggesting significant topology. SOC effects create an indirect band gap of 0.11 eV. After SOC, the CBM stays at the M point, but no longer crosses the fermi level.

Investigating the fatbands in the third row of Figure 12, SbAsH_2_ clearly has no band inversion, similarly to BiAsH_2_ in Figure 6g. Figure 12g shows that the conduction bands are dominated entirely by Sb-5p orbitals. The valence bands are dominated solely by As-4p orbitals. SbAsO_2_ has some orbital mixing, as shown in Figure 12h. The lowest conduction band is largely dominated by Sb-5p orbitals, but is dominated by As-4p orbitals around the Γ point. As shown in Figure 12i, the fatbands of SbAsS_2_ around the fermi level are not dominated by either p orbital. The lower conduction band is dominated by Sb-5p orbitals at high energy levels. The upper conduction band is dominated by As-4p orbitals at high energy levels.

**Figure 13:**
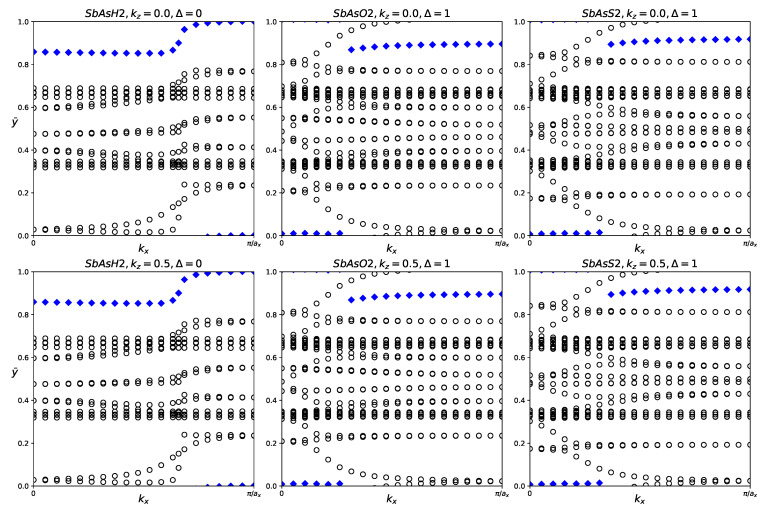
Hybrid Wannier charge centers for functionalized SbAsX_2_ monolayers (X = H, O, S). The open circles represent hybrid Wannier charge centers. The first row is at the surface k_z_ = 0. The second row is at the surface k_z_ = 0.5. The Δ indicates the Z_2_ invariant—0 represents trivial topology, while 1 represents nontrivial topology.

For each structure, we calculated the Z_2_ invariant by tracking the evolution of the hybrid Wannier charge centers, as shown in Figure 13. Indeed, SbAsH_2_ does not have significant topological nature. SbAsO_2_ and SbAsS_2_ were calculated to have Z_2_ = 1, indicating nontrivial topology.

SbAsH_2_ is a trivial semiconductor. SbAsO_2_ and SbAsS_2_ are small band gap topological insulators.

### 3.5. BiSbX_2_ (X = H, O, S)

**Figure 14:**
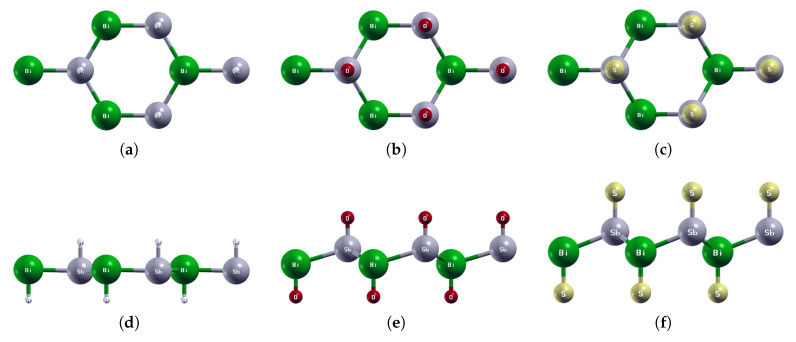
Optimized atomic structures of BiSbX_2_ (X = H, O, S). Displayed here are the (**a**–**c**) top and (**d**–**f**) side views of a 2 × 2 × 1 supercell of (**a**,**d**) BiSbH_2_; (**b**,**e**) BiSbO_2_; (**c**,**f**) BiSbS_2_. Each atom is labeled with its atomic symbol. The green, gray, white, red, and grayish-yellow atoms represent Bi, Sb, H, O, and S, respectively.

**Figure 15:**
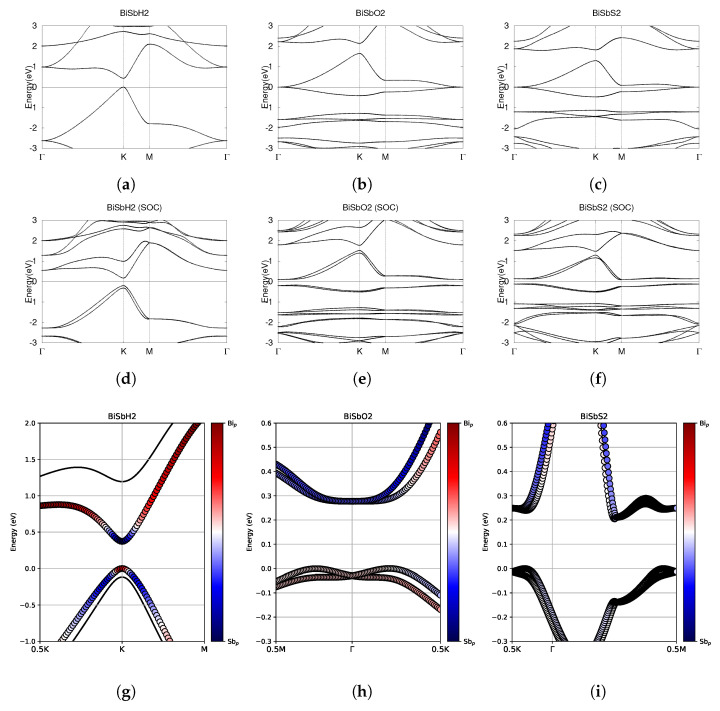
Electronic band structures of functionalized monolayers BiSbH_2_, BiSbO_2_, and BiSbS_2_. (**a**–**c**) The top row displays the bands without SOC. (**d**–**f**) The middle row displays the bands with SOC. (**g**–**i**) The bottom row shows the fatbands, with SOC. The fermi energy level position from the DFT calculation is set to 0.

The optimized atomic structures of BiSbX_2_ (X = H, O, S) are shown in Figure 14. All four monolayers are buckled honeycomb structures, appearing as a zigzag pattern from the side view. Each structure has four atoms per unit cell. The lattice constants, buckling heights, and electronic band structures of the functionalized BiSbX_2_ monolayers are shown in Table 7 and Figure 15.

As shown in Figure 14d, BiSbH_2_ buckles inwards. BiSbO_2_ and BiSbS_2_ buckle outwards. Similarly to BiAsH_2_ and SbAsH_2_ discussed previously, BiSbH_2_ has a very small buckling height of 0.08 Å compared to 0.91 Å for BiSbO_2_ and 1.16 Å for BiSbS_2_, as listed in Table 7.

Investigating the electronic band structure, Figure 15a,d reveals that BiSbH_2_ has a direct band gap at the K point both with and without SOC. Before SOC, BiSbH_2_ has a band gap of 0.43 eV. After SOC, the band gap is reduced to 0.37 eV. BiSbO_2_ has VBM and CBM degenerate at the Γ point, forming a Dirac point. SOC effects breaks the degeneracy at Γ, indicating significant topological nature. With SOC, the conduction bands are twofold degenerate at the Γ point, as shown in Figure 15e and with greater detail in Figure 15h. The valence bands are also twofold degenerate at the Γ point. After SOC, BiSbO_2_ has an indirect band gap of 0.28 eV, as listed in Table 7. As shown in Figure 15c, BiSbS_2_ has VBM and CBM degenerate at the Γ point, forming a Dirac point. SOC effects breaks the degeneracy at Γ, indicating significant topological nature. With SOC, the conduction bands are twofold degenerate at the Γ point. The valence bands are also twofold degenerate at the Γ point. After SOC, the CBM is between the K and M points. When SOC effects are taken into account, BiSbS_2_ has an indirect band gap of 0.20 eV, as listed in Table 7.

Investigating the fatbands in the third row of Figure 15, BiSbH_2_ has an incredibly clear band inversion at the Γ point, indicating significant topological nature. BiSbO_2_ has some orbital mixing, as shown in Figure 15h. The conduction bands are largely dominated by Bi-6p orbitals, but Sb-5p orbitals still appear in the conduction band. Similarly, the valence bands are largely dominated by Sb-5p orbitals, but Bi-6p orbitals still appear in the valence band. The fatbands of BiSbS_2_ are shown in Figure 15i. The lower conduction band is only dominated by Sb-5p orbitals at high energy levels. The upper conduction band is dominated by Bi-6p orbitals. The valence bands are unclear, with some orbital mixing.

**Figure 16:**
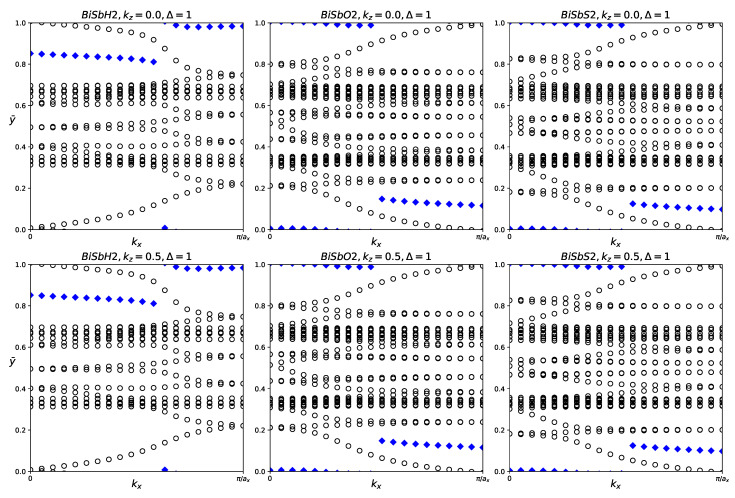
Hybrid Wannier charge centers for functionalized BiSbX_2_ monolayers (X = H, O, S). The open circles represent hybrid Wannier charge centers. The first row is at the surface k_z_ = 0. The second row is at the surface k_z_ = 0.5. The Δ indicates the Z_2_ invariant—0 represents trivial topology, while 1 represents nontrivial topology.

The fatbands of BiSbH_2_ clearly indicate topological nature, BiSbO_2_ and BiSbS_2_ are unclear. We tracked the hybrid Wannier charge centers for each material and calculated the Z_2_ invariant, listed in Table 7. The evolution of the hybrid Wannier charge centers can be seen in Figure 16. Indeed, BiSbH_2_ has Z_2_ = 1, confirming its topological nature. BiSbO_2_ and BiSbS_2_ both have Z_2_ = 1, indicating significant topology.

BiSbH_2_, BiSbO_2_, and BiSbS_2_, are all topological insulators.

## 4. Conclusions

We performed first-principles calculations to investigate the atomic structure, electronic band structure, and topological nature of binary VA-VA monolayers BiAs, AsP, SbAs, and BiSb, and functionalized BiAsX_2_, AsPX_2_, SbAsX_2_, and BiSbX_2_ (X = H, O, S). Our calculations predict 8 room-temperature topological insulators with small band gaps: BiAsO_2_, BiAsS_2_, AsPO_2_, SbAsO_2_, SbAsS_2_, BiSbH_2_, BiSbO_2_, and BiSbS_2_. These materials all hold great potential for applications in room-temperature spintronics and other fields. Our results also predict 1 topological semimetal: AsPS_2_.

We believe our theoretical results reflect real-world properties, and possible directions for future work include further exploration of functionalized Group VA-VA binary monolayers to fill the gap of room-temperature topological insulators. However, theoretical prediction is known to be limited, and we hope our predicted room-temperature topological insulators will inspire experimental research. Topological materials would contribute to practical applications within quantum computing, spintronics, and nanoelectronics.

## Figures and Tables

**Table 1:** Electron configurations and radius cutoffs in angstroms of the atoms used in current calculations.

Element	Electron Configuration	Radius Cutoff (Å)
P	[Ne]3s^2^3p^3^	1.01
As	[Ar]4s^2^3d^10^4p^3^	1.11
Sb	[Kr]5s^2^4d^10^5p³^3^	1.27
Bi	[Xe]6s^2^4f^14^5d^10^6p^3^	1.28
H	1s^1^	0.53
O	[He]2s^2^2p^4^	0.75
S	[Ne]3s^2^3p^4^	1.01

**Table 2:** Converged kinetic energy cutoff, k-point grid, and vacuum height for each structure.

Structure	Kinetic Energy Cutoff (Ha)	k-Point Grid	Vacuum Height (Å)
BiAs	21	10 × 10 × 1	17
BiAsH_2_	21	8 × 8 × 1	19
BiAsO_2_	21	8 × 8 × 1	21
BiAsS_2_	21	8 × 8 × 1	25
AsP	14	10 × 10 × 1	13
AsPH_2_	21	10 × 10 × 1	22
AsPO_2_	21	8 × 8 × 1	19
AsPS_2_	21	10 × 10 × 1	24
SbAs	14	10 × 10 × 1	15
SbAsH_2_	21	10 × 10 × 1	19
SbAsO_2_	21	8 × 8 × 1	20
SbAsS_2_	21	8 × 8 × 1	25
BiSb	21	10 × 10 × 1	18
BiSbH_2_	21	8 × 8 × 1	19
BiSbO_2_	21	6 × 6 × 1	20
BiSbS_2_	21	8 × 8 × 1	25

**Table 3:** Current and previous other calculations of BiAs, AsP, SbAs, BiSb. *a* (Å) is the lattice constant; *h* (Å) is the buckling height; *E_g_* (eV) and $E_{g}^{SOC}$ (eV) are the band gaps calculated without and with SOC, respectively. Z_2_ is the topological invariant. 1 represents nontrivial topology, 0 indicates trivial topology.

Materials	BiAs		AsP		SbAs		BiSb	
Properties	Current	Other	Current	Other	Current	Other	Current	Other
*a* (Å)	3.98	3.982 [45], 4.00 [28,31]	3.48	3.45 [46], 3.46 [31]	3.86	3.87 [24], 3.86 [31,35]	4.23	4.255 [47], 4.24 [28,31,48]
*h* (Å)	1.56	1.532 [45], 1.56 [28,31]	1.34	1.33 [46], 1.32 [31]	1.52	1.52 [24,35], 1.51 [31]	1.69	1.69 [28,31,47,48]
*E_g_* (eV)	1.12		1.70	1.82 [46]	1.39	1.47 [35]	1.00	0.95 [47], 0.96 [48]
$E_{g}^{SOC}$ (eV)	0.75	0.70 [45]	1.61		1.20	1.27 [35]	0.40	0.37 [47], 0.36 [48]
Z_2_	0		0		0		0	

**Table 4:** The calculated lattice parameters and band gaps of BiAsX_2_ (X = H, O, S). *a* (Å) is the lattice constant; d (Å) is the bond length between the two subscripted atoms; *h* (Å) is the buckling height; *E_g_* (eV) and *$E_{g}^{SOC}$* (eV) are the band gaps calculated without and with SOC, respectively. Z_2_ is the topological invariant. 1 represents nontrivial topology, 0 indicates trivial topology.

System	*a* (Å)	$d_{\mathrm{Bi}-\mathrm{As}}$ (Å)	$d_{\mathrm{Bi}-X}$ (Å)	$d_{\mathrm{As}-X}$ (Å)	*h* (Å)	*E_g_* (eV)	$E_{g}^{\mathrm{SOC}}$ (eV)	Z_2_
BiAsH_2_	5.05	2.92	1.81	1.54	0.04	0.79	0.05	0
BiAsO_2_	4.99	3.03	1.93	1.65	0.96	0	0.21	1
BiAsS_2_	4.78	2.99	2.33	2.06	1.15	0	0.09	1

**Table 5:** The calculated lattice parameters and band gaps of AsPX_2_ (X = H, O, S). *a* (Å) is the lattice constant; d (Å) is the bond length between the two subscripted atoms; *h* (Å) is the buckling height; *E_g_* (eV) and $E_{g}^{SOC}$ (eV) are the band gaps calculated without and with SOC, respectively. Z_2_ is the topological invariant. 1 represents nontrivial topology, 0 indicates trivial topology.

System	*a* (Å)	$d_{\mathrm{Bi}-\mathrm{As}}$ (Å)	$d_{\mathrm{Bi}-X}$ (Å)	$d_{\mathrm{As}-X}$ (Å)	*h* (Å)	*E_g_* (eV)	$E_{g}^{\mathrm{SOC}}$ (eV)	Z_2_
AsPH_2_	3.44	2.52	1.69	1.48	1.54	0	0	0
AsPO_2_	4.08	2.60	1.64	1.49	1.09	0	0.05	1
AsPS_2_	4.02	2.59	2.04	1.92	1.15	0	0	1

**Table 6:** The calculated lattice parameters and band gaps of SbAsX_2_ (X = H, O, S). *a*(Å) is the lattice constant; d(Å) is the bond length between the two subscripted atoms; *h*(Å) is the buckling height; *E_g_*(eV) and $E_{g}^{SOC}$(eV) are the band gaps calculated without and with SOC, respectively. Z_2_ is the topological invariant. 1 represents nontrivial topology, 0 indicates trivial topology.

System	*a* (Å)	$d_{\mathrm{Bi}-\mathrm{As}}$ (Å)	$d_{\mathrm{Bi}-X}$ (Å)	$d_{\mathrm{As}-X}$ (Å)	*h* (Å)	*E_g_* (eV)	$E_{g}^{\mathrm{SOC}}$ (eV)	Z_2_
SbAsH_2_	4.93	2.85	1.73	1.53	0.06	0.35	0.04	0
SbAsO_2_	4.74	2.93	1.83	1.64	1.05	0	0.12	1
SbAsS_2_	4.53	2.88	2.23	2.05	1.20	0	0.11	1

**Table 7:** The calculated lattice parameters and band gaps of BiSbX_2_ (X = H, O, S). *a*(Å) is the lattice constant; d(Å) is the bond length between the two subscripted atoms; *h*(Å) is the buckling height; *E_g_*(eV) and $E_{g}^{SOC}$(eV) are the band gaps calculated without and with SOC, respectively. Z_2_ is the topological invariant. 1 represents nontrivial topology, 0 indicates trivial topology.

System	*a* (Å)	$d_{\mathrm{Bi}-\mathrm{As}}$ (Å)	$d_{\mathrm{Bi}-X}$ (Å)	$d_{\mathrm{As}-X}$ (Å)	*h* (Å)	*E_g_* (eV)	$E_{g}^{\mathrm{SOC}}$ (eV)	Z_2_
BiSbH_2_	5.36	3.10	1.81	1.73	0.08	0.43	0.37	1
BiSbO_2_	5.32	3.20	1.94	1.84	0.91	0	0.28	1
BiSbS_2_	5.07	3.15	2.34	2.25	1.16	0	0.20	1

## Data Availability

The original contributions presented in this study are included in the article. Further inquiries can be directed to the corresponding author and additional specific data can be provided upon reasonable request.

## Abbreviations

The following abbreviations are used in this manuscript:

DFT	Density Functional Theory
PAW	Projector Augmented Wave
HWCC	Hybrid Wannier Charge Centers
SOC	Spin-Orbit Coupling
TSM	Topological Semimetal
TI	Topological Insulator
VBM	Valence Band Maximum
CBM	Conduction Band Minimum
